# Evaluation of Major Autohemotherapy (MAH) in Psoriasis Patients Using Serum Inflammatory Markers

**DOI:** 10.3390/jcm15020485

**Published:** 2026-01-08

**Authors:** Seçil Soylu, Nazlı Şensoy, Nurhan Doğan, Halit Buğra Koca, Tülay Köken

**Affiliations:** 1 Department of Dermatology and Venereology, Division of Internal Medical Sciences, Faculty of Medicine, Afyonkarahisar Health Sciences University, Afyonkarahisar 03030, Türkiye; 2 Department of Family Medicine, Division of Internal Medical Sciences, Faculty of Medicine, Afyonkarahisar Health Sciences University, Afyonkarahisar 03030, Türkiye; nazli.sensoy@afsu.edu.tr; 3 Department of Biostatistics and Medical Informatics, Division of Basic Medical Sciences, Faculty of Medicine, Afyonkarahisar Health Sciences University, Afyonkarahisar 03030, Türkiye; nurhan.dogan@afsu.edu.tr; 4 Department of Medical Biochemistry, Division of Basic Medical Sciences, Faculty of Medicine, Afyonkarahisar Health Sciences University, Afyonkarahisar 03030, Türkiye; bugra.koca@afsu.edu.tr (H.B.K.); tulay.koken@afsu.edu.tr (T.K.)

**Keywords:** ozone therapy, psoriasis, inflammation, biomarkers, tnf-α, il-1β, high-sensitivity c-reactive protein (hs-crp), sialic acid, siglec-14, treatment

## Abstract

**Background/Objectives**: Psoriasis is a chronic, inflammatory, systemic skin disease. Although topical and systemic drugs with proven effectiveness are used in the treatment, ozone therapy is also applied as a treatment option based on clinical personal experience and with limited published knowledge. In this project, the aim was to evaluate the effectiveness of major ozone therapy in psoriasis patients together with biomarkers in serum. **Methods**: A total of 26 psoriasis patients and 19 healthy controls were included in the study. The disease severity was evaluated by the psoriasis area severity index score and grouped as mild, moderate/severe. Serum tumor necrosis factor alpha (TNF-α), interleukin 1-beta (IL-1β), high-sensitivity C-reactive protein (Hs-CRP), sialic acid, and Sialic acid binding Ig-like Lectin-14 (Siglec-14) levels were investigated in controls and psoriasis patients. **Results**: Psoriasis area severity index (PASI) score decreased significantly in psoriasis patients after ozone autohemotherapy application (*p* < 0.005). The values of IL-1β, sialic acid, and Siglec-14 after treatment in healthy subjects were statistically significantly higher than in psoriasis patients. It was found that Hs-CRP and Siglec-14 decreased in all patients after treatment, Hs-CRP decreased more significantly in mild psoriasis patients, and Siglec-14 decreased in both mild and moderate-severe groups (*p* < 0.05). **Conclusions**: Our research results suggest that ozone autohemotherapy has clinical efficacy in psoriasis patients, inflammation also has a role in the mechanism of action, and its effectiveness in treatment can be evaluated with inflammation markers.

## 1. Introduction

Psoriasis is a chronic inflammatory skin disease characterized by a complex pathogenesis involving not only keratinocyte proliferation but also interactions between the immune system, metabolic pathways, microbiota, and environmental factors [1,2]. Recent studies have revealed that psoriasis is not limited to skin involvement but is a systemic disease with significant effects on quality of life, comorbidities, and treatment responses [3]. The mutual interaction between keratinocytes, dendritic cells, and especially Th17 cells is one of the fundamental mechanisms underlying the onset and chronic course of the disease. The stimulation of keratinocytes by cytokines such as IL-17, IL-22, and TNF-α results in the release of various chemokines and inflammatory mediators; this cycle contributes to the formation and persistence of psoriatic plaques [4].

In addition to the immune response, changes at the metabolic and genomic levels also play a role in psoriasis flare-ups and in determining disease severity [5]. Alongside genetic predisposition and epigenetic changes, new biomarkers identified in omics studies offer potential benefits in monitoring disease progression and selecting treatments [2,6,7]. Furthermore, imbalances in the gut microbiota have been shown to be associated with psoriatic inflammation through Treg functions, NF-κB activation, and short-chain fatty acid levels [8].

Clinically, psoriasis is characterized by scaly plaques, erythema, and thickened lesions, and is evaluated in conjunction with comorbidities such as metabolic syndrome and cardiovascular diseases associated with systemic inflammation [9]. Therefore, the treatment approach is not limited to controlling skin findings; it also aims to suppress inflammation and improve quality of life. Current treatments offer a wide range of options, from topical medications to systemic agents, biological therapies, and new targeted agents [10].

Interest in alternative and complementary therapies continues. Ozone therapy shows potential anti-inflammatory effects through mechanisms such as activation of the antioxidant defense system, suppression of the NF-κB signaling pathway, and reduction in Th17 cell activity [11,12]. Various studies have reported that topical ozone applications reduce the inflammatory response in psoriasis lesions, support keratinocyte differentiation, and suppress NF-κB-mediated processes [11,12,13,14]. However, most of these studies focus on topical ozone application; the number of studies evaluating major ozone autohemotherapy (MAH) and serum biomarkers together in psoriasis is quite limited.

In this context, the current study aims to evaluate the clinical efficacy of MAH in psoriasis patients and to examine this efficacy through inflammatory biomarkers such as TNF-α, IL-1β, Hs-CRP, sialic acid, and Siglec-14 (BT Lab Bioassay Technology Laboratory, Shanghai, China). The limited number of studies in the literature that evaluate MAH in psoriasis in terms of both clinical outcomes and biomarker levels highlights the importance of this research.

## 2. Materials and Methods

This study was supported by the Scientific Research Projects Unit of the Faculty of Medicine, Afyonkarahisar Health Sciences University (Project No: 19.TIP.009) and approved by the Non-Interventional Clinical Research Ethics Committee of the Faculty of Medicine, Atatürk University (No: 2022/571, Date: 18 August 2022). The necessary approval was obtained from the Afyonkarahisar Provincial Health Directorate (Commission Decision No: 2022/24, Date: 7 July 2022).

During the preparation of this manuscript, ChatGPT version 5 was used to verify the consistency between in-text citations and the bibliographic references in the reference list.

### 2.1. Patients and Controls Included in the Study

This study was designed as a case–control study. The study was conducted between 20 July 2022 and 5 September 2022 at the Traditional Complementary Medicine Unit of Afyonkarahisar State Hospital, Afyonkarahisar Health Sciences University Health Application and Research Hospital Dermatology and Venereology Clinic, and Family Medicine Clinic. Patients aged 18–60 who presented to the Dermatology and Family Medicine outpatient clinics, had a prior diagnosis of psoriasis based on physical examination and/or histopathological evaluation, and were not using systemic drug therapy were included in the study. Verbal and written consent were obtained from each individual participating in the study. The G Power software package (GPower v3.1.9) used to determine the number of participants to be included in the study for a two-factor experimental design involving repeated measurements on a single factor. Here, Type I error (alpha) = 0.05, effect size (d) = 0.03, and power = 80% were set. Accordingly, the total sample size was determined to be 50. However, considering that participants might drop out of the study, an additional 10% was added, and 55 individuals were included in the study. During the study, some participants withdrew. The study was completed with a total of 45 individuals, including 26 patients and 19 control group members.

### 2.2. Inclusion Criteria

1. Patients aged 18–60 with a diagnosis of psoriasis who consent to participate in the study and who present to the Dermatology and Venereology Clinic or the Family Medicine Clinic at the Health Application and Research Center of Afyonkarahisar Health Sciences University.
2. Healthy individuals aged 18–60 who presented to the Dermatology and Venereal Diseases and Family Medicine clinics, had no systemic diseases, were not taking any medications, and agreed to participate in the study.

### 2.3. Exclusion Criteria

1. Individuals who have received systemic psoriasis treatment within the last 6 months
2. Glucose-6-phosphate dehydrogenase deficiency (favism)
3. Pregnancy
4. Obesity
5. Acute alcohol intoxication and alcohol users
6. Recent myocardial ischemia
7. Hemorrhage in any organ
8. Immune system disease
9. Hyperthyroidism
10. Severe anemia
11. Diabetes mellitus
12. Severe myasthenia gravis
13. Neoplastic
14. Liver and kidney disease
15. Those who have recently undergone surgery
16. Use of diuretics
17. Familial hypercholesterolemia
18. Hormone replacement therapy
19. Ozone allergy
20. Those who exercise excessively
21. Those who have used vitamins and anti-inflammatory drugs in the last 3 months

### 2.4. Data Collection, Scales and Tests Used, Major Ozone Autohemotherapy

A personal data form was completed for all participants in the study, which included questions about age, gender, marital status, educational status, occupation, smoking habits, alcohol consumption, comorbidities, and duration of psoriasis. Serum levels of TNF-α, IL-1β, SA, Siglec-14, and Hs-CRP were measured before and after MAH. The PASI scale was used to calculate psoriasis severity and evaluate response to treatment.

PASI: Each area is assessed individually: head (h), upper extremities (u), trunk (t), and lower extremities (l). The severity of lesions covering VYA (A), induration (I), erythema (E), and desquamation (D) is scored on a scale of 0–4. If there are no symptoms, zero is entered; if symptoms are mild, one; if moderate, two; if pronounced, three; if severe, four. When calculating the surface area, if the percentage of involvement is less than 10%, it is scored as one; between 10% and 29%, it is scored as two; between 30% and 49%, it is scored as three; between 50% and 69%, it is scored as four; between 70% and 89%, it is scored as five; and if it is more than 90%, it is scored as six. The body coefficient is also given according to the region represented in the formula. Accordingly, the head, upper extremity, trunk, and lower extremity are included in the calculation with fixed coefficients of 0.1, 0.2, 0.3, and 0.4, respectively [15].

As seen in the formula:

- PASI = 0.1(Eh + Ih + Dh)Ah + 0.2(Eu + Iu + Du)Au + 0.3(Et + It + Dt)At + 0.4(El + Il + Dl)Al

The area score is multiplied by the body coefficient representing it, and the sum of the desquamation, induration, and erythema scores in the relevant area. The sum of the separate severity values for each body region gives the PASI score. The maximum score is 72 [15,16]. Based on the PASI score obtained, the disease is classified as mild (0–9), moderate (10–19), and severe (20 and above).

In the study, the rule of 10 was used to define psoriasis as “severe.” Accordingly, patients were grouped as mild (PASI score below 9) or moderate/severe (PASI score of 10 or above). Psoriasis patients were divided into three groups: “before ozone therapy,” “after 5 courses,” and “after 8–10 courses of treatment.” PASI scores were reassessed in these three groups and analyzed as PASI initial, PASI second, and PASI final, respectively.

Participants’ blood samples were collected from the antecubital vein between 8:00 and 9:00 a.m. after a 12 h fast, totaling 5–6 mL, into heparinized vacuum tubes for serum. The collected blood samples were centrifuged at 2000 rpm for 10 min. The upper portion of the plasma was pipetted into Eppendorf tubes and stored at −80 degrees Celsius. The samples were analyzed in the Department of Medical Biochemistry laboratory.

### 2.5. Measurement of Serum TNF-α, IL-1β, hs-CRP, Sialic Acid, and Sialic Acid Binding Ig-like Lectin-14 Levels

Measurement of serum TNF-α, IL-1β, hs-CRP, SA, and Siglec-14 levels was performed using BT-LAB brand Human TNF-α, IL-1β, CRP, SA, and Siglec-14 ELISA kits (BT Lab Bioassay Technology Laboratory, Shanghai, China). Absorbance readings were performed using a ChemWell 2910 brand ELISA reader (Awareness Technology, Inc., Martin Hwy, Palm City, FL, USA). Results were given as ng/L for TNF-α, pg/mL for IL-1β, ng/mL for Hs-CRP, mg/dL for SA, and ng/L for Siglec-14.

### 2.6. Major Ozone Autohemotherapy Application

MAH was administered twice a week to the patient and control group for a total of 8–10 courses at the Traditional and Complementary Medicine Unit of Afyon State Hospital. For this procedure, the patient’s vein was first accessed using a Terumo butterfly 19/21 G needle (Terumo Corporation, Tokyo, Japan) made of ozone-resistant silicone material. Subsequently, 100 mL of blood was drawn from the transfusion set made of ozone-resistant silicone material and placed into a sterile, single-use, negative-pressure, citrate-containing glass bottle specifically manufactured for ozone therapy. The citrate contained in the glass bottle prevents the blood from clotting. A filtered set placed in the special area for ozone application at the entrance of the glass bottle was used to deliver a 100 mL volume of oxygen/ozone mixture at a medium dose of 20 μg/mL concentration from the ozone generator (SALUTEM medical ozone device) into the bottle at a low speed, and the mixture was treated for approximately 2–3 min. The glass bottle was then lifted and hung on the IV pole. The IV set lock was opened, and the patient’s “own blood,” which had been treated with ozone, was returned through the same transfusion set within approximately 10–15 min. The patient and control group included in the study were initially given an oxygen/ozone mixture at a concentration of 20 μg/mL, and the ozone/oxygen concentration was gradually increased for each application according to the patient, reaching a maximum of 60 μg/mL.

### 2.7. Data Analysis

The Statistical Package for the Social Sciences (SPSS) 20.0 software package was used for data analysis. In the descriptive analysis section, categorical variables were presented using numbers and percentages, while continuous variables were presented using means and standard deviations. The chi-square test was used to compare categorical variables. The Kolmogorov–Smirnov test was applied to determine the distribution of all variable groups in secondary comparisons. Repeated Measures Analysis of Variance was used to compare three or more dependent groups, and the Bonferroni test was used to identify significantly different groups. The Student-*t* test was used to compare two independent groups, and the Paired *t*-test was used to compare two dependent groups. The Mann–Whitney U test and the Wilcoxon T test were used for variables that did not have a normal distribution. Statistical significance was accepted as *p* < 0.05 for all analyses.

## 3. Results

The study included 26 (57.8%) psoriasis patients and 19 (42.2%) healthy controls. The patient and control groups had similar characteristics in terms of gender, smoking status, and marital status. A significant difference was found in terms of educational status, with the patient group having a lower educational level than the controls (*p* < 0.05). There was a difference in age between the two groups, with a higher number of patients in the under-40 age group (*p* < 0.05). When individuals with psoriasis were examined according to their PASI scores for mild and moderate/severe disease, it was found that most patients had mild disease. The socio-demographic characteristics of the patients and their PASI scores are summarized in Table 1.

The mean duration of psoriasis in patients was 142.6 ± 111.67 months, ranging from 12 to 420 months. It was found that PASI scores decreased significantly between pre-ozone therapy and after the fifth session, as well as between pre-treatment and post-treatment, but there was no significant difference between the values after the fifth MAH and after the final MAH.

Before and after ozone therapy, differences in serum biomarker levels between patients and healthy controls were examined. No statistically significant difference was found between patients and controls before treatment; however, IL-1β, SA, and Siglec-14 levels were found to be statistically significantly higher in healthy individuals than in psoriasis patients after treatment (Table 2).

When comparing the pre- and post-treatment values of inflammatory markers in all patients regardless of psoriasis severity, Hs-CRP and Siglec-14 were found to decrease with treatment, while no significant differences were observed for other markers. In healthy controls, a significant decrease in IL-1β was observed. Except for the increase in TNF-α in healthy individuals, when all markers were evaluated within their own groups in both patients and controls, they were observed to have decreased, although no statistically significant difference was found with treatment (Table 2).

There was no difference between the disease severity and marker values before and after treatment in the patient group (*p* > 0.05) (Table 3).

The relationship between the severity of the disease and differences in markers before and after treatment was compared. Following treatment, Hs-CRP decreased only in patients with mild disease, while Siglec-14 decreased in both the mild and moderate/severe groups (*p* < 0.05). Although no statistically significant difference was found, it was observed that all biomarker values decreased after ozone therapy in both severity levels of the disease, except for the increase in SA and IL-1β (Table 3).

## 4. Discussion

There are only a limited number of studies in the literature examining the clinical efficacy and mechanism of action of ozone therapy in psoriasis in humans. Most studies focus on topical ozone application. The number of studies examining the clinical effectiveness of MAH in psoriasis, particularly in relation to biomarkers involved in its pathogenic mechanisms, remains very limited [13,17,18,19,20,21].

To the best of our knowledge, this study is the first to evaluate the clinical efficacy of MAH therapy in psoriasis using serum levels of the disease biomarkers TNF-α, Hs-CRP, SA, IL-1β, and Siglec-14.

Psoriasis is considered to be a chronic inflammatory and systemic disease mediated by autoimmune and immune factors, in addition to genetic factors. In recent years, its frequent association with other inflammatory diseases and the high levels of systemic inflammation markers found in severe cases have raised the possibility that it may be a systemic disease [22,23,24,25,26]. Furthermore, it has been suggested that chronic inflammation in psoriasis contributes to the development of metabolic and vascular disorders. Consequently, studies have focused particularly on comorbidities associated with psoriasis, the common mechanisms of these diseases, the efficacy of systemic psoriasis treatment on comorbidities, and biomarkers [23,24,27].

The relationship between ozone therapy and biomarkers in certain diseases has been investigated, and it has been shown that local ozone therapy inhibits IL-1β and Hs-CRP expression in patients with periodontitis using topical ozone therapy [28]. It has been reported that local ozone therapy after implant surgery accelerates tissue wound healing, reduces tissue inflammation, and shows a significant decrease in CRP levels in patients receiving ozone therapy compared to the control group, both between sessions and at the end of treatment [29]. Similarly, topical ozone therapy has been shown to significantly reduce TNF-α and IL-1β levels in doxorubicin-induced skin necrosis [30]. A review on the use of topical ozone therapy in psoriasis lesions stated that it is a beneficial treatment alternative when used as a complement to drug therapy. The reasons for its success may be related to its antibacterial effect, providing immune regulation, its strong antioxidant effect, altering the epigenetics of the disease, and the restoration of the skin through the regulation of the microbiome of damaged skin [13].

The findings of our study indicate that MAH therapy resulted in a significant reduction in both Hs-CRP and Siglec-14 levels. The significant decrease in Hs-CRP levels following treatment suggests that MAH may affect not only the dermatological manifestations of psoriasis but also its systemic inflammatory component. Given that elevated Hs-CRP levels are associated with increased cardiovascular risk in psoriasis, this reduction may potentially confer long-term benefits regarding comorbidities. The marked decrease in Siglec-14 levels may be related to the regulation of the innate immune response; MAH’s suppression of the inflammatory response via the TLR/NF-κB axis may have contributed to clinical improvement by balancing the more aggressive immune response seen in Siglec-14-positive phenotypes.

Gao et al. demonstrated that ozonated oil application in humans and rats with psoriasis may promote recovery from the disease by inducing the differentiation of basal keratinocytes while suppressing excessive proliferation [21]. Small-scale clinical studies have shown that autohaemotherapy, when used in combination with topical ozonated oil applications, reduces PASI scores and alleviates clinical symptoms; these findings suggest that the treatment produces an effect consistent with its predicted anti-inflammatory mechanisms [12]. Similarly, in our study, PASI scores decreased in patients with treatment, and there was a general decrease in biomarkers.

In this study, the relatively higher levels of certain inflammatory markers found in healthy controls after treatment suggest that psoriasis may have a heterogeneous immune response pattern in terms of systemic inflammation. It has been reported that inflammation is mostly confined to the local tissue level, especially in mild or stable phases of the disease, and may not be reflected to the same extent in serum levels. Furthermore, these findings observed in healthy individuals may be related to conditions independent of psoriasis, such as subclinical inflammation, genetic variations affecting the immune response, environmental exposures, or differences in the microbiota. Nevertheless, the observation of a marked decrease in the psoriasis group after treatment supports the regulatory effect of MAH on the systemic inflammatory response.

The effect of systemic ozone therapy has also been investigated in diseases other than psoriasis. In patients with chronic inflammatory conditions, diabetic foot, neurodegenerative diseases, and COVID-19, a significant decrease in Hs-CRP, TNF-α, and IL-1β levels was found to correlate with clinically significant improvement [17,31,32,33,34].

Psoriasis is a chronic and recurrent condition, so not only clinical efficacy but also sustainability and cost considerations are important. MAH can be considered a complementary option in situations where access to high-cost biological treatments is limited. Considering the potential social benefits, such as symptom control, improved quality of life, reduced workforce loss, and decreased healthcare visits, MAH may find a broader application in psoriasis management.

One of the mechanisms that increases cellular damage in psoriasis is oxidative stress. Ozone therapy can activate cellular antioxidant defense systems with controlled oxidative stimulation and balance the oxidative load by increasing antioxidant response components such as glutathione, superoxide dismutase, and catalase via Nrf2. Therefore, the effect of MAH in psoriasis may be related not only to the suppression of inflammatory cytokines but also to the re-regulation of oxidative stress. When NF-κB pathways are activated by numerous inflammatory cytokines in psoriatic epidermis, it results in hyperproliferation of keratinocytes. Activation of toll-like receptor 2 (TLR2) in keratinocytes can lead to nuclear translocation of NF-κB and release of proinflammatory cytokines. Numerous studies have demonstrated that the TLR2/NF-κB signaling pathway intensifies the inflammatory response by inducing the release of numerous inflammatory factors in psoriasis lesions. This leads to abnormal proliferation and differentiation of keratinocytes due to proinflammatory cytokines. As a result, the production of chemokines and adhesion molecules increases, contributing to the persistence of the neutrophil-mediated inflammatory cycle in plaques. MAH therapy is thought to improve psoriasis by reducing inflammatory factors associated with psoriasis in the systemic circulation [18,20].

In another study conducted by Zeng et al. on experimental mice, they found that MAH reduced the severity of psoriasis and decreased TNF-α in peripheral blood CD4+ T cells, and that the expression of inflammatory cytokines associated with psoriasis decreased with topical ozone therapy [35]. Authors have noted that, due to psoriasis being a chronic, recurrent skin disease that occurs alongside systemic inflammatory diseases, ozone therapy may control the progression of the disease through different mechanisms by inhibiting the inflammatory response in skin lesions. First, it has been reported that ozone therapy reduces the activation of the TLR2/NF-κB pathway, thereby inhibiting microorganisms colonizing the lesion surface and thus reducing the production of microorganisms and their components, namely pathogen-associated molecular patterns. Second, the activation of the antioxidant system in the body, such as Nrf2, by ozone can antagonize NF-κB-mediated inflammatory responses. Third, the oxygen produced by ozone therapy can improve the hypoxic environment of psoriatic skin lesions and inhibit hypoxia-induced inflammatory responses [18,20].

No articles have been found in the literature evaluating CRP, SA, and Siglec values in psoriasis using MAH. It can be stated that Hs-CRP and Siglec-14 were significantly reduced in patients with MAH, regardless of the severity of psoriasis, and that other inflammatory values were also reduced, although not statistically significant. We also found that the treatment reduced biomarkers more significantly in patients with psoriasis than in controls, and that biomarkers were higher in patients with severe psoriasis than in those with mild disease.

It cannot be said that MAH is a completely side-effect-free application. The literature reports mild side effects such as temporary headache, fatigue, and irritation at the injection site. Furthermore, due to the short duration of existing studies, it is unclear whether MAH provides long-term remission. As there is no strong evidence regarding the duration of the effect after treatment ends, long-term follow-up studies should be conducted to evaluate the sustainability of the effect.

The limited follow-up and treatment period and the small number of cases are limitations in this study.

In conclusion, our study has demonstrated that MAH may be effective in the treatment of psoriasis and can be used in combination with conventional therapies. Furthermore, although no significant difference in biomarker levels was initially detected between healthy individuals and psoriasis patients, the fact that biomarker levels were significantly lower in psoriasis patients compared to the control group after treatment can be explained by the following possibilities:

1. Biomarkers whose role in the pathogenesis of the disease has not been conclusively proven were evaluated.
2. Since psoriasis is an inflammatory process, inflammatory parameters decreased more markedly with MAH application, even if no difference was observed initially.
3. The results obtained suggest that the disease is affected not only by inflammation but also by other pathogenic mechanisms.

Biomarkers and their treatments in psoriasis and comorbidities, as well as changes in these biomarkers, remain an area of ongoing research. We believe that randomized controlled trials investigating the efficacy, safety, and mechanisms of action of MAH in psoriasis are necessary, and we hope that our study contributes to the literature in this field.

## Figures and Tables

**Table 1:** Participants’ socio-demographic characteristics and PASI scores.

Categories	Patient (n = 26; %57.8)		Control (n = 19; %42.2)		
	n	%	n	%	*p*
Gender					
Female	9	42.9	12	57.1	0.11
Male	17	70.8	7	29.2	
Age (years)					
<40	19	73.1	6	31.6	0.014
≥40	7	26.9	13	68.4	
Marital status					
Married	20	76.9	13	68.4	0.767
Single	6	23.1	6	31.6	
Education					
≤High school	14	53.8	1	5.3	0.01
>High school	12	46.2	18	94.7	
Smoking					
Yes	12	46.2	8	42.1	1.00
No	14	53.8	11	57.9	
PASI					
Mild	16	61.5			
Moderate/Severe	10	38.5			

*p*: Chi-square test.

**Table 2:** Evaluation of inflammatory markers in patients and healthy controls before (‘First’) and after (‘Last’) ozone therapy.

		Patient		Control		
		Median (IQR)	Median (Min–Max)	Median (IQR)	Median (Min–Max)	*p* *
TNF-α						
	First	59.7 (17.8)	36.4–452.3	73.2 (90.1)	10.3–439.2	0.077
	Last	54.5 (28.5)	30.6–417.1	75.7 (119.9)	11.3–466.8	0.198
*p* **		0.304		0.243		
IL-1β						
	First	701.9 (271.20)	197.4–3763.0	1271.0 (1470.8)	59.8–3376.0	0.071
	Last	685.9 (309.65)	423.8–3234.0	1237.0 (1766.8)	100.2–3756.0	0.020
*p* **		0.694		0.049		
Hs-CRP						
	First	8.7 (4.2)	6.1–26.4	11.3 (8.5)	4.9–22.5	0.232
	Last	8.3 (3.4)	5.7–17.4	9.9 (6.0)	3.0–26.4	0.103
*p* **		0.026		0.059		
Sialic acid						
	First	48.3 (162.1)	25.6–187.7	55.0 (26.0)	10.9–147.3	0.241
	Last	45.7 (93.0)	27.5–120.5	54.8 (56.2)	17.3–186.0	0.023
*p* **		0.112		0.212		
Siglec-14						
	First	653.8 (459.2)	530.4–3188.0	744.4 (461.0)	484.5–1710.0	0.646
	Last	637.5 (185.3)	496.3–1575.0	729.9 (705.4)	447.0–3240.0	0.015
*p* **		0.011		0.601		

*p* *: Mann–Whitney U Test; *p* **: Wilcoxon *t* Test.

**Table 3:** Relationship between differences in marker values before (‘First’) and after (‘Last’) treatment in psoriasis patients and disease severity.

		PASI Initial					PASI Last				
		Mild (n = 16)		Severe (n = 10)			Mild (n = 19)		Severe (n = 7)		
		Median (IQR)	Range (Min–Max)	Median (IQR)	Range (Min–Max)	*p* *	Median (IQR)	Range (Min–Max)	Median (IQR)	Range (Min–Max)	*p* *
TNF-α											
	First	60.6 (36.7)	43.5–452.3	57.4 (24.0)	36.4–108.0	0.370	59.9 (15.4)	37.3–452.3		36.4–108.0	0.885
	Last	54.4 (29.0)	32.0–417.1	53.4 (823.5)	30.6–126.1	0.562	53.3 (31.9)	30.6–417.1		36.7–126.1	0.751
*p* **		0.215		0.959			0.212		1.000		
IL-1β											
	First	674.4 (260.3)	418.8–3763.0	772.9 (433.9)	197.4–1806.0	0.635	671.4 (278.4)	197.4–3763.0	783.7 (367.9)	468.5–1806.0	0.099
	Last	685.9 (319.8)	423.8–3234.0	692.9 (330.4)	432.5–1217.0	0.874	697.8 (305.2)	423.8–3234.0	805.8 (334.5)	521.5–1217.0	0.370
*p* **		0.877		0.575			0.841		0.237		
Hs-CRP											
	First	8.6 (7.0)	6.1–26.4	9.2 (4.6)	6.1–25.4	0.673	8.5 (2.9)	6.1–26.3	9.07 (12.4)	6.1–25.4	0.665
	Last	8.1 (3.0)	5.7–15.1	8.3 (9.1)	6.9–17.4	0.155	7.97 (2.7)	5.7–16.8	8.6 (8.2)	6.9–17.4	0.099
*p* **		0.039		0.386			0.044		0.398		
Sialic acid											
	First	45.3 (48.7)	25.6–187.7	50.9 (39.6)	34.6–147.9	0.370	48.3 (30.0)	25.6–187.7	53.1 (92.5)	34.6–147.9	0.236
	Last	41.3 (15.5)	27.5–109.9	52.3 (35.9)	37.5–120.5	0.051	41.5 (19.0)	27.5–115.4	47.4 (14.7)	38.1–120.5	0.214
*p* **		0.163		0.508			0.469		0.091		
Siglec-14											
	First	685.0 (467.8)	530.4–3188.0	643.4 (273.4)	548.4–1100.0	0.370	716.1 (468.5)	530.4–3188.0	633.2 (112.0)	535.7–1100.0	0.193
	Last	644.9 (151.7)	540.9–1209.0	593.5 (328.4)	496.3–1575.0	0.370	657.3 (178.5)	500.0–1575.0	546.7 (132.3)	496.3–1031.0	0.053
*p* **		0.039		0.139			0.044		0.043		

*p* *: Mann-Whitney U Test; *p* **: Wilcoxon *t* Test.

## Data Availability

The raw data supporting the conclusions of this article will be made available by the authors on request.

## Abbreviations

The following abbreviations are used in this manuscript:

CRP	C-reactive protein
ELISA	Enzyme-linked immunosorbent assay
HDL-C	High-density lipoprotein cholesterol
Hs-CRP	High-sensitivity C-reactive protein
IFN-γ	Interferon gamma
IL-1β	Interleukin 1 beta
IL-6	Interleukin 6
IL-17	Interleukin 17
IL-17A	Interleukin 17A
IL-22	Interleukin 22
IL-23	Interleukin 23
IQR	Interquartile range
MAH	Major autohemotherapy
NF-κB	Nuclear factor kappa-light-chain-enhancer of activated B cells
Nrf2	Nuclear factor erythroid 2–related factor 2
OMICS	Global “omics” platforms (genomics, proteomics, metabolomics, etc.)
PASI	Psoriasis Area and Severity Index
PPAR-γ	Peroxisome proliferator-activated receptor gamma
SA	Sialic acid
Siglec-14	Sialic acid-binding immunoglobulin-like lectin 14
SPSS	Statistical Package for the Social Sciences
TCA	Tricarboxylic acid cycle
Th17	T helper 17 cells
TLR2	Toll-like receptor 2
TNF-α	Tumor necrosis factor alpha
Treg	Regulatory T cells
